# Replacement Condition Detection of Railway Point Machines Using an Electric Current Sensor

**DOI:** 10.3390/s17020263

**Published:** 2017-01-29

**Authors:** Jaewon Sa, Younchang Choi, Yongwha Chung, Hee-Young Kim, Daihee Park, Sukhan Yoon

**Affiliations:** 1 Department of Computer and Information Science, Korea University, Sejong 30019, Korea; sjwon92@korea.ac.kr (J.S.); ycc4477@korea.ac.kr (Y.C.); dhpark@korea.ac.kr (D.P.); 2 Department of Applied Statistics, Korea University, Sejong 30019, Korea; starkim@korea.ac.kr; 3 Sehwa R&D Center, Techno 2-ro, Yuseong-gu, Daejeon 34026, Korea; shy5406@hanmail.net

**Keywords:** maintenance engineering, railway point machine, electric current shape analysis, replacement condition monitoring

## Abstract

Detecting replacement conditions of railway point machines is important to simultaneously satisfy the budget-limit and train-safety requirements. In this study, we consider classification of the subtle differences in the aging effect—using electric current shape analysis—for the purpose of replacement condition detection of railway point machines. After analyzing the shapes of after-replacement data and then labeling the shapes of each before-replacement data, we can derive the criteria that can handle the subtle differences between “does-not-need-to-be-replaced” and “needs-to-be-replaced” shapes. On the basis of the experimental results with in-field replacement data, we confirmed that the proposed method could detect the replacement conditions with acceptable accuracy, as well as provide visual interpretability of the criteria used for the time-series classification.

## 1. Introduction

Fault diagnosis methods [1,2] have been widely applied in many applications such as roller bearings [3], gearboxes [4], wind turbines [5], and tool wear [6] by using various sensor signals such as vibration [7]. Recently, fault diagnosis results have been reported for railway applications [8,9,10,11,12,13,14,15] because maintenance of the constituent components of the railway is important to ensure that the train can be safely driven. The railway point machine (RPM)—consisting of a motor, reduction gear, several bearings, derive-detection rods, and switches [8]—is an especially important component that changes the traveling direction of a train. RPM uses an electric motor to move a switch blade from one position to another, making electric current-based fault diagnosis methods feasible. For example, classification methods using support vector machine (SVM) with the discrete wavelet transform (DWT) [16] and uniform scaling [17] have been reported as a diagnostic of the failure of RPMs. These methods work by extracting features of the electric current signal obtained from the RPMs. In addition, an electric current-based fault diagnosis method using dynamic time warping (DTW) [18] has been proposed to manage the variation in durations of RPM movement. Recently, sound signal-based fault diagnosis method [19] has reported as a possible means to achieve this goal.

In this study, we focus on the aging effect for the replacement of RPMs, rather than fault diagnosis. In safety- or mission-critical applications such as railway transportation, maintenance of key components such as RPM should be conducted carefully [20]. That is, when a fault is identified with fault diagnosis, it should be repaired immediately [21]. However, aging effects progress slowly, and thus, the replacement decision needs to be carefully conducted to detect the subtle differences between “does-not-need-to-be-replaced” and “needs-to-be-replaced” (i.e., due to the slowly progressing effects). In general, there are guidelines for RPM replacement such as the operation period (e.g., more than 10 years) or the number of accumulated movements (e.g., more than 100,000 movements) before replacement. However, in practice, these guidelines are not followed, and it is even difficult to obtain in-field replacement data due to the relatively long operation period. Furthermore, we also need to consider the operational condition of each RPM, such as weather/train-load/train-speed variation [21], for detecting aging effects. For example, RPMs that can operate well owing to fair operational conditions may be replaced later, or those that operate poorly owing to harsh operational conditions may not be earlier, according to the simple guideline. Therefore, we must determine whether an RPM needs to be replaced or not depending on the in-field operational conditions, for simultaneously satisfying the budget-limit and RPM-safety requirements. In practice, however, keeping all these operational conditions of each RPM in mind is almost impossible.

In this study, we assume the electric current shape of each RPM reflects its operational condition and propose a classification method that employs electric current shape analysis in order to determine whether an RPM needs to be replaced or not. In particular, we focus on the aging effect for the replacement of RPMs, and thus our method should also handle the more subtle differences in aging effects rather than fault diagnosis. After analyzing the shapes of “after-replacement” (i.e., brand new) RPMs and then labeling the shapes of each “before-replacement” (i.e., used for more than 10 years) RPM, we can automatically derive the criteria that can handle the subtle differences between “does-not-need-to-be-replaced” and “needs-to-be-replaced” shapes in the before-replacement RPMs. To the best of our knowledge, this is the first report on the RPM replacement-condition detection problem that shows how to classify the shapes accurately, with in-field replacement data, (not laboratory simulated data).

The rest of this paper is organized as follows: Section 2 describes the proposed replacement condition detection method with some background concept. The experimental results with in-field replacement data are presented in Section 3, followed by the conclusions in Section 4.

## 2. Method for Detecting Replacement Condition

As in [16,17,18], the electric current shape of an RPM movement is an acceptable choice for RPM fault diagnosis. However, the previous methods used for detecting the fault condition may not be able to detect the replacement condition. For example, Kim et al. [18] proposed a fault diagnosis method using DTW to detect the fault condition. The method had the advantage of detecting the abnormal shape in the electric current data without a training step. Even with the flexible-distance measure (e.g., DTW), however, this DTW method may not be able to distinguish the shapes between does-not-need-to-be-replaced and needs-to-be-replaced, owing to the more subtle differences in the aging effects than fault diagnosis.

**Figure 1:**
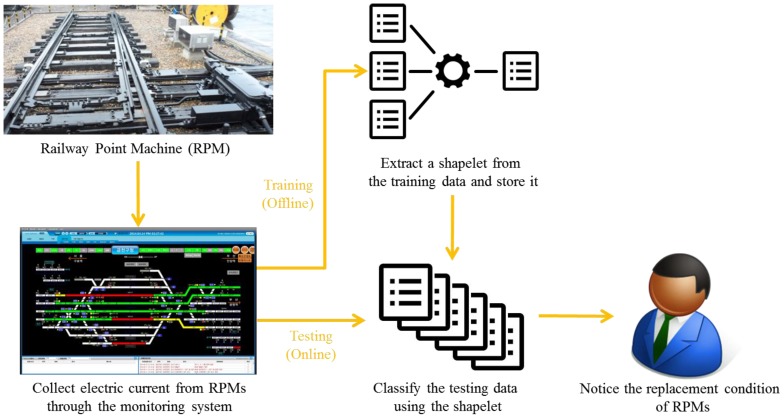
Overall structure of the proposed method for analyzing electric current shapes.

Figure 1 shows the overall structure of the proposed method. After collecting both before- and after-replacement data obtained from the RPM monitoring system, we label the before-replacement data into two classes: does-not-need-to-be-replaced and needs-to-be-replaced. Then, features minimizing the within-class distance and maximizing the between-class distance are extracted in an offline training phase. Finally, an electric current shape of each RPM movement is analyzed with the extracted features in order to detect a replacement condition of the RPM in an online testing phase. 

For example, the after-replacement data obtained from the in-field replacement data in Korea had some common electric current pattern although different RPMs were made from different manufacturers. Based on this common pattern, we labeled the electric current patterns of in-field before-replacement data (with an operation period of more than 10 years) with the help of maintenance staff. Some of the before-replacement data had a similar pattern with the common pattern of the after-replacement data (i.e., “does-not-need-to-be-replaced”). However, most of the before-replacement data had subtle differences from the common pattern of the after-replacement data (i.e., “needs-to-be-replaced” due to the aging effects). Thus, we need to develop an automatic decision method that can classify the before-replacement data into “does-not-need-to-be-replaced” or “needs-to-be-replaced”. 

Note that no fault has been reported for the before-replacement data measured, and each RPM in the measurement has different operational conditions as shown in Table 1. Note also that some properties such as the length, peak, or average value of the before-replacement data are different from those of the after-replacement data. If we use these properties such as [22], however, we cannot explain the difference in the operational condition in the before-replacement data (e.g., different number of accumulated movements, in addition to weather/maintenance difference). In order to focus on the shape itself, we first apply normalization to both before- and after-replacement data, as in [23,24]. 

In this study, we consider a shape-based method for analyzing the normalized electric current patterns in RPMs for detecting the subtle differences in the aging effect. The shapelet algorithm [23] has been used for time-series shape classification and extracts a subsequence called a shapelet that consists of features minimizing the within-class distance and maximizing the between-class distance, through training. The extracted shapelet can then be used to compare testing time-series data with it. Thus, the shapelet algorithm has the advantage of the execution time of the classification over the DTW, which compares the testing time-series data with the whole sequence of the reference data. In addition, it also has the advantage of acquiring a shapelet that can support intuitive interpretation through time-series data. Therefore, the shapelet algorithm can detect subtle differences in the aging effect. 

With the shapelet algorithm, a shapelet should be extracted from the training time-series data in order to solve the two-class (i.e., class *D_a_*: not-need-to-be-replaced or class *D_b_*: need-to-be-replaced) classification problem. Let us assume the training dataset *D* has *n* labeled time series data. *D* includes the time-series data *T* of length *m* where *T* = *t*_1_, *t*_2_, …, *t_m_*. The subsequence *S* of length *l* can then be extracted from a starting point *p* in *T* where length and Z normalization are applied. The minimum distance is then found among the distance results by using Equation (1). Here, $S_{l}^{*}$ is the set of all possible subsequences of length *l* that can be extracted from *T*, and *S*′ is one among all the possible subsequences.

(1) $$S_{l}^{*}=\left\{S_{p}^{l}\;\mathrm{of}\;T_{p}^{l}|T_{p}^{l}=t_{p},t_{p+1},\ldots ,t_{p+l-1}\right\}\;dist\left(S\prime ,T\right)=\min\left(dist\left(S_{l}^{*},T_{p}^{l}\right)\right)$$

Afterwards, the entropy is calculated for splitting the classes in *D*. Equation (2) shows the entropy to search the split, where *n_c_*, *n_i_*, and *p_i_* are the number of classes in *D*, the number of objects in class *i*, and class probability in *D*, respectively.

(2) E(D)=∑i=1nc−pilog(pi), pi=nin

Before obtaining information gain for split *sp*, *D* needs to be separated into *D_a_* and *D_b_* using the distance threshold that separates two small datasets. *sp* can then be calculated from Equation (3), where *n_a_* and *n_b_* are the number of time series data in *D_a_* and *D_b_*, respectively.

(3) $$I\left(sp\right)=E\left(D\right)-\frac{n_{a}}{n}E\left(D_{a}\right)-\frac{n_{b}}{n}E\left(D_{b}\right)$$

*sp* can be used to obtain the separation gap by calculating two time-series data from *D_a_* and *D_b_*. Equation (4) shows the calculation of the separation gap using *sp*:

(4) $$gap\left(sp\right)=\frac{1}{n_{a}}\underset{T_{a}\in D_{a}}{\sum }dist\left(S\prime ,T_{a}\right)-\frac{1}{n_{b}}\underset{T_{b}\in D_{b}}{\sum }dist\left(S\prime ,T_{b}\right)$$

Finally, a shapelet can be determined as a split with the maximum information gain through the gap in the datasets.

After extracting the shapelet, each of the testing data is classified by using a traditional decision tree through the distance between the testing data and the shapelet extracted. The details of the shapelet algorithm can be found in [23,24].

Before the shapelet algorithm is applied, length and Z normalization are applied to electric current time-series data in order to the focus on the “local” shape. A shapelet can then be extracted by training the normalized data through the shapelet algorithm. Finally, the distance between the shapelet and the testing data is calculated using the Euclidean distance to measure their similarity. 

**Figure 2:**
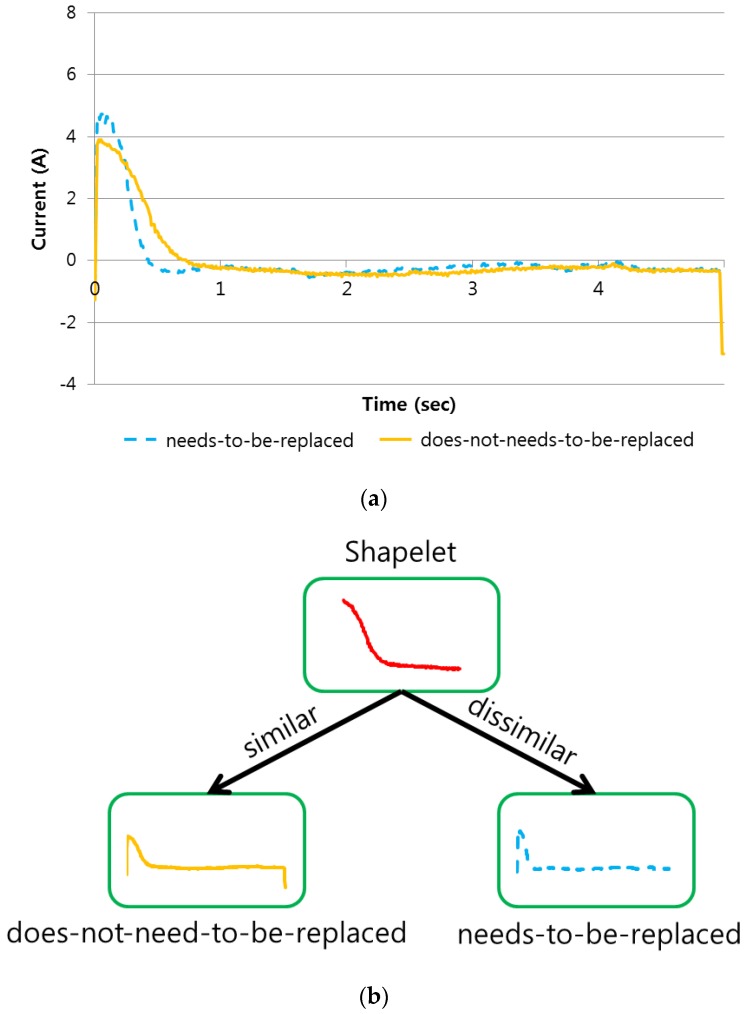
Extraction of a shapelet and the corresponding decision tree: (**a**) Subtle differences in the aging effect in the before-replacement data; (**b**) A decision tree using the shapelet extracted.

Figure 2a shows one example of the does-not-need-to-be-replaced and needs-to-be-replaced pairs in the before-replacement data. As shown in Figure 2a, the normalized before-replacement data exhibits a more clear difference at the beginning between the does-not-need-to-be-replaced and needs-to-be-replaced shapes. Note that the normalized does-not-need-to-be-replaced shape in the before-replacement data is very similar to the normalized after-replacement shape (i.e., we denote the shape as does-not-need-to-be-replaced). With the help of maintenance staff, the shapelet shown in Figure 2b was validated as an acceptable feature to detect the aging effect. That is, in safety- or mission-critical applications such as railway transportation, the domain expert knowledge is very important. The extracted shapelet was consistent with the domain expert knowledge (i.e., the criteria labeling the before-replacement data into two classes), and this “visual interpretability” is the one of the main advantages of the shapelet algorithm [23] over typical classification methods such as SVM, neural network, and DTW. 

In fact, the case shown in Figure 2b is similar to the case for electrocardiogram (ECG) data [24]. The shapelet extracted with ECG data captured the criteria for the two-class classification problem, with 99.4% accuracy (compared to 79.7% accuracy with DTW). In addition, the ECG criteria were verified by a domain expert (i.e., a USC cardiologist stated that the delayed t-wave was the only medically significant difference between the two classes, and the shapelet algorithm identified this part as the shapelet).

## 3. Experimental Results

### 3.1. Experimental Data

Table 1 describes the properties for the in-field data of RPMs collected at seven stations in Korea during operation periods before replacement. Some RPMs were replaced despite their few accumulated movements, meaning that the typical replacement guidelines are not followed in practice. For this reason, a method for detecting the replacement condition of RPMs is required to consider the shape itself.

Our experimental environment was as follows: Intel Core^®^ i5-4670 3.40 GHz, 8 GB RAM, and Windows 7 Professional 64-bit (Microsoft, Redmond, WA, USA). We used the fast shapelet algorithm [24] to extract a shapelet more rapidly than with the original shapelet algorithm [23]. We obtained the in-field measurement data for analysis (captured at a sampling rate of 100 Hz) from 39 RPMs (seven stations in Korea), as shown in Table 1.

**Figure 3:**
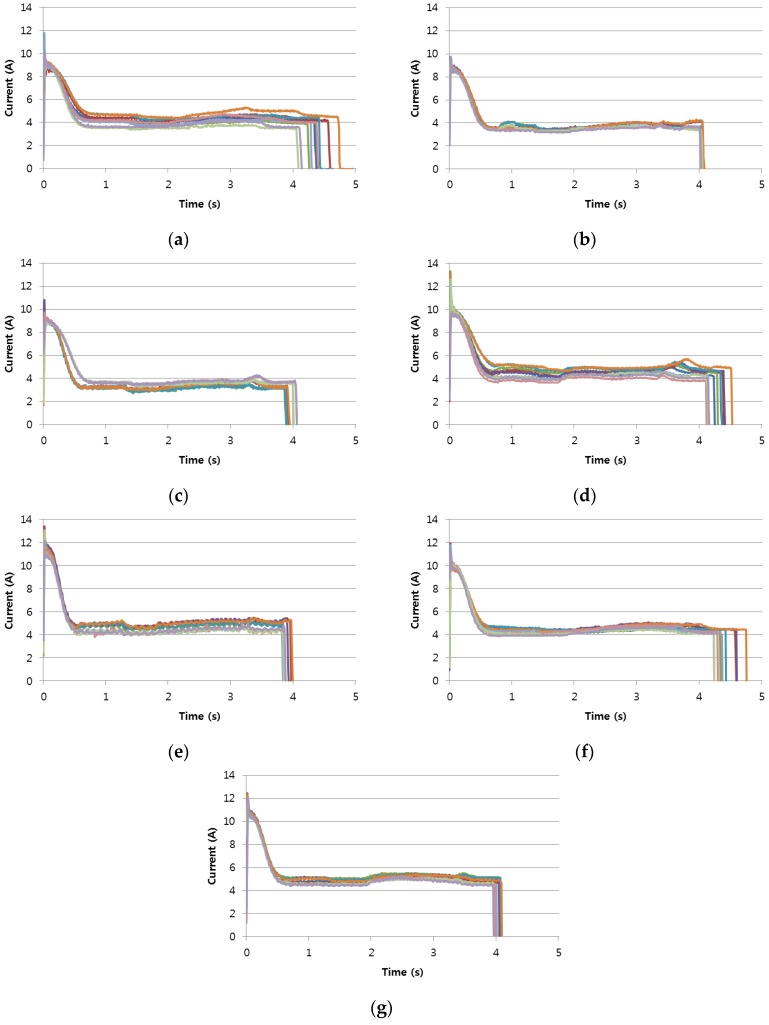
Electric current shapes of RPMs measured during one year after replacement: (**a**–**g**) describe the after-replacement RPM shapes in each station.

The obtained data consisted of two types: before-replacement and after-replacement. As shown in Figure 3, the after-replacement data had some common electric current pattern although different RPMs were made from different manufacturers. For readability, Figure 3 shows only the first 10 RPM movements measured in each station from the large number of movements measured. With this common pattern, we can extract a shapelet for the purpose of replacement condition detection.

**Figure 4:**
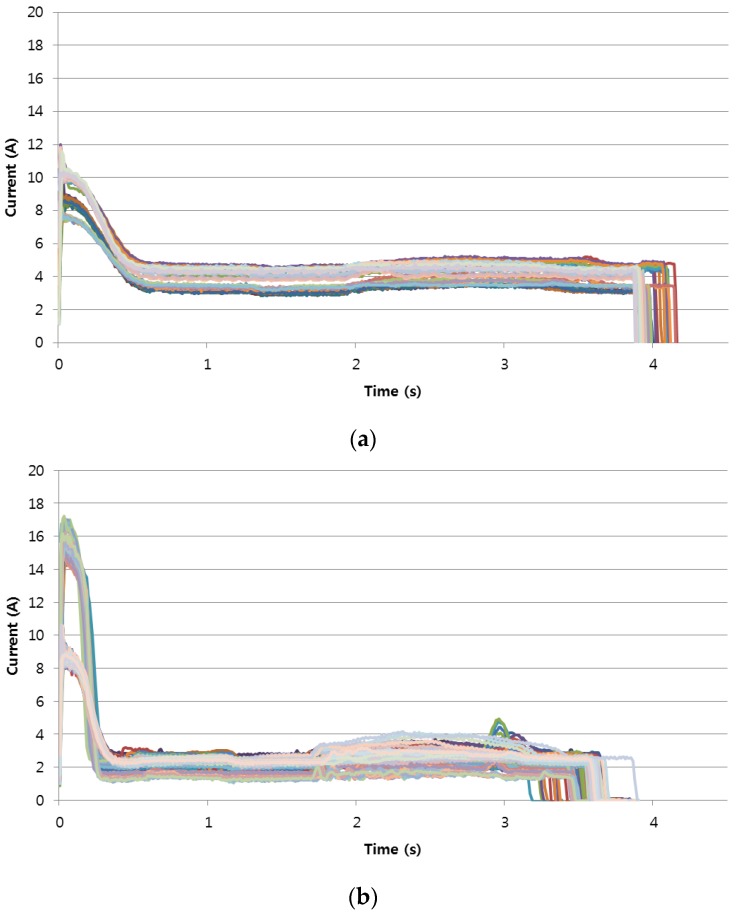
Electric current shapes of RPMs measured during one year before replacement: (**a**) does-not-need-to-be-replaced; (**b**) needs-to-be-replaced.

Next, with the help of maintenance staff, the before-replacement data were categorized into two classes: does-not-need-to-be-replaced and needs-to-be-replaced. As we can see in Figure 4, there are subtle differences between the two classes, in addition to some variations within each class. Note that, through the Length and Z normalization [23,24], the variations within each class can be reduced and we can focus on the shape itself.

### 3.2. Classification Results

For evaluating the proposed method, all of the data (i.e., 913 shapes) obtained from each station were separated equally as training and testing data. In the before-replacement data, the ratio of does-not-need-to-be-replaced to needs-to-be-replaced was approximately 1/9. To solve the data imbalance between the two classes [25], we merged the after-replacement data (i.e., does-not-need-to-be-replaced) with the before-replacement data so that the ratio of does-not-need-to-be-replaced to needs-to-be-replaced became approximately 11/9 (i.e., balanced scenario), in addition to the imbalanced scenario (i.e., both training and testing were conducted with the before-replacement data only). Further, “positive” refers to the minority class (i.e., needs-to-be-replaced in the balanced scenario and does-not-need-to-be-replaced in the imbalanced scenario) in the accuracy evaluation. We then conducted a two-fold cross-validation with the training and testing data to analyze the classification performance of the proposed method. In addition, the validation was conducted five times to verify the variation in the experimental results by using random sampling in the labeled data [26]. During the experiments, two parameters, max-length and min-length, which determine the length of a shapelet, were set to 200 and 1, respectively. 

To evaluate the performance of the proposed method, we compared it with the DTW-based method [18]. For a fair comparison, Z-normalization was also applied to the DTW-based method (because DTW itself can handle the length difference, it does not need length normalization). In addition, to evaluate the effect of selecting the most prominent (i.e., visually interpretable) subsequence of the shapelet-based method (called Shapelet-Subsequence for the purposes of explanation), we intentionally set the length of a shapelet as the length of the sequence and measured its performance (called Shapelet-Fullsequence for the purposes of explanation). In a strict sense, the Shapelet-Fullsequence method is not a shapelet-based method, because it does not select the visually interpretable subsequence. Rather, it can be regarded as a hybrid between Shapelet-Subsequence and DTW, and the performance of Shapelet-Fullsequence can help explain the difference between Shapelet-Subsequence and DTW. Table 2 summarizes the analysis methods used in this section.

**Figure 5:**
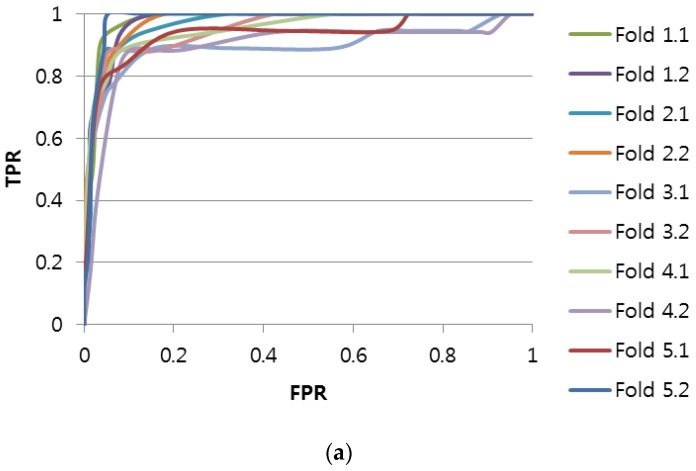

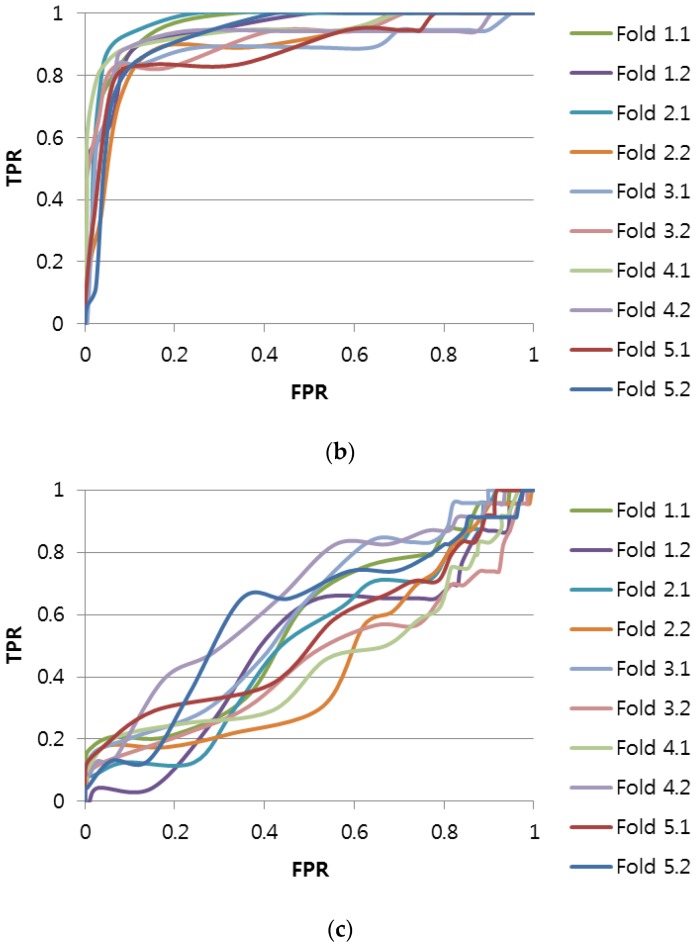
Receiver operating characteristic (ROC) curves for the imbalanced scenario using two-fold cross-validation with five repetitions: (**a**) ROC of Shapelet-Subsequence; (**b**) ROC of Shapelet-Fullsequence; (**c**) ROC of DTW.

**Figure 6:**
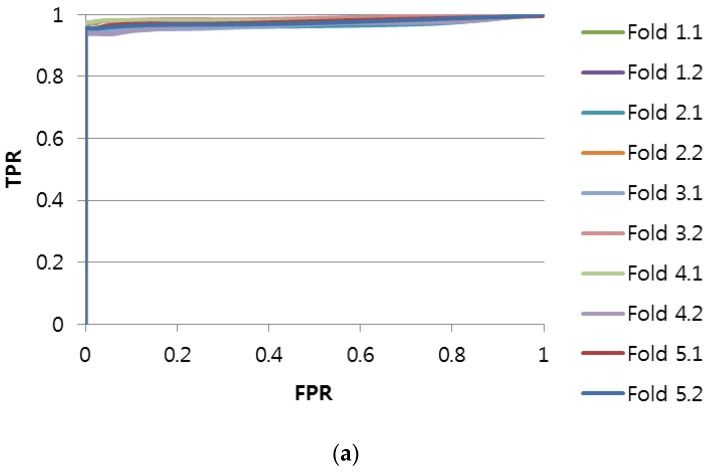

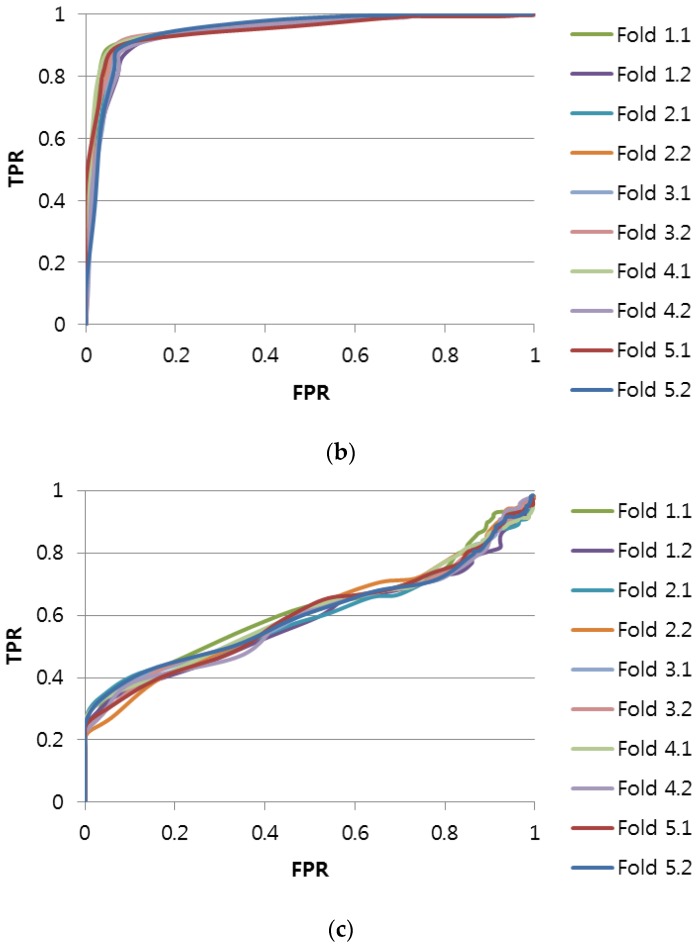
Receiver operating characteristic (ROC) curves for the balanced scenario using two-fold cross-validation with five repetitions: (**a**) ROC of Shapelet-Subsequence; (**b**) ROC of Shapelet-Fullsequence; (**c**) ROC of DTW.

The accuracy can be evaluated by computing True Positive (*TP*), False Positive (*FP*), True Negative (*TN*), and False Negative (*FN*) [27]. Figure 5 shows the accuracy as a receiver operating characteristic (ROC) curve for the imbalanced scenario, whereas Figure 6 shows the accuracy as an ROC curve for the balanced scenario. We also show the accuracy computed as the area under ROC (AUROC) and the execution time of each method in Table 3 and Table 4, respectively.

### 3.3. Discussion

From Figure 5, we can see that the accuracy of the Shapelet-Subsequence is high enough for practical application (ROC curves of the Shapelet-Subsequence drop sharply at FPR = 0 similar to an ideal ROC curve). Interestingly, the accuracy of the Shapelet-Fullsequence was much higher than that of DTW, although it was lower than that of the Shapelet-Subsequence. This can be explained by the fact that the aging effect causes subtle differences in the length-normalized electric current shape. That is, both the Shapelet-Subsequence and Shapelet-Fullsequence methods could detect the subtle differences effectively, whereas the DTW method was too flexible for the accuracy to deteriorate. 

In Table 4, the training time of the shapelet-based method is for shapelet extraction, whereas the training time of the DTW-based method is for representative sequence extraction. Although the shapelet extraction in the offline training phase is time consuming, the subsequence comparison with the Euclidean distance in the online testing phase is much faster than the full-sequence comparison with the DTW distance. Note that the testing time consists of normalization and comparison times, and the testing times of all three methods were faster than the RPM movement period (i.e., approximately 5 s) to make real-time execution possible. 

Although accurate cost-effectiveness requires complicated remaining useful life (RUL) estimation [28] and is not within the main scope of this study, we can approximately compute the cost-effectiveness of the Shapelet-Subsequence method. For example, by setting the *True Positive Rate* to 0.95 (i.e., most of the needs-to-be-replaced conditions correctly classified as needs-to-be-replaced) in Figure 6, the Shapelet-Subsequence method could obtain a zero *False Positive Rate* (i.e., all the does-not-need-to-be-replaced conditions correctly classified as does-not-need-to-be-replaced). That is, if the replacement cost of each RPM is 15,000,000 Korean Won, then the shapelet-based electric current analysis method can prevent the unnecessary replacement cost of 60,000,000 Korean Won (corresponding to four not-need-to-be-replaced RPMs from the total of 39 replaced RPMs) in the operation-period-based replacement. This cost saving is estimated with the experimental data. If we extrapolate the saved portion (i.e., 10%) to the scale of the entire country (i.e., more than 1000 RPMs are replaced annually), then the estimated saving is approximately 1,500,000,000 Korean Won annually.

In fact, the main contribution of this study is that we developed an electric current monitoring system, installed it near the in-field RPMs in Korea, collected the electric current shapes of the RPMs for more than two years, and automatically analyzed the before- and after-replacement data. Before this study, the domain experts in a Korean railway company did not check the electric current shapes of the before- and after-replacement RPMs. Since the Shapelet-Subsequence method could provide a domain expert “visual interpretability” of the criteria used for the time-series classification (compared to the typical classification methods), the domain experts agreed that the automatic analysis method could improve the reliability of each RPM under a limited budget. Note again that, in safety- or mission-critical applications such as railway transportation, domain experts are very conservative, and providing the visual interpretability of the proposed technique is important to be accepted by the domain expert. Furthermore, we believe this study can help the conservative railway community to move toward the emerging *Industry 4.0* [29].

## 4. Conclusions

Management of RPMs is vital to prevent serious accidents such as train derailments. In particular, it is generally difficult to obtain in-field RPM replacement data, and thus automatic detection of in-field replacement conditions has not been reported yet, to the best of our knowledge.

In this study, we proposed an electric current shape-based analysis method for detecting the replacement condition of RPMs. The proposed method first extracts a shapelet that can detect the subtle differences in the aging effect, and then distinguishes the does-not-need-to-be-replaced shape from the needs-to-be-replaced shape by using the extracted shapelet. On the basis of the experimental results with in-field replacement data (i.e., operated data for more than 10 years, not laboratory simulated data), the classification accuracy of the proposed method for detecting the replacement condition was much higher than the accuracy of the flexible-distance measure (e.g., DTW) based method. Furthermore, the proposed method can provide domain experts with the visual interpretability of the criteria used for time-series classification.

## Figures and Tables

**Table 1:** Properties of RPMs in each station.

Station Type	# of RPMs Replaced	# of RPMs Measured	Operation Period before Replacement (Years)	# of Accumulated Movements before Replacement	# of Movements Measured for Analysis
A	15	14	12	1284–33,272	406
B	13	3	12–14	653–19,391	47
C	17	7	12–14	12,875–107,927	141
D	2	1	10–13	11,442–137,370	24
E	7	5	12–16	5778–391,141	113
F	5	4	13–14	5209–82,795	64
G	8	5	14–17	436–108,600	118

**Table 2:** Summary of the analysis methods.

Method	Normalization	Comparison	Distance
Shapelet-Subsequence	Length and Z	Subsequence	Euclidean
Shapelet-Fullsequence	Length and Z	Full-sequence	Euclidean
DTW [18]	Z	Full-sequence	DTW

**Table 3:** Average accuracy (AUROC) of the analysis methods.

Method	Imbalanced Scenario	Balanced Scenario
Shapelet-Subsequence	0.95	0.97
Shapelet-Fullsequence	0.92	0.94
DTW [18]	0.53	0.60

**Table 4:** Average execution time of the analysis methods.

Method	Training (Unit: Second)	Testing (per RPM Movement) (Unit: Millisecond)
Shapelet-Subsequence	35.54	0.921
Shapelet-Fullsequence	0.21	0.994
DTW [18]	0.12	8.308
